# Role of Endocrine System in the Regulation of Female Insect Reproduction

**DOI:** 10.3390/biology10070614

**Published:** 2021-07-02

**Authors:** Muhammad Zaryab Khalid, Sajjad Ahmad, Patrick Maada Ngegba, Guohua Zhong

**Affiliations:** 1 Key Laboratory of Natural Pesticide and Chemical Biology, Ministry of Education, South China Agricultural University, Guangzhou 510642, China; zaryabkhalid0003@hotmail.com (M.Z.K.); sajjadahmadbhatti@outlook.com (S.A.); patrickmaadangegba@gmail.com (P.M.N.); 2 Termite Management Laboratory, Department of Entomology, University of Agriculture Faisalabad, Faisalabad 38000, Pakistan; 3 Sierra Leone Agricultural Research Institute, Tower Hill, Freetown P.M.B 1313, Sierra Leone

**Keywords:** endocrinology, ecdysteroids, 20-hydroxyecdysone, juvenile hormone, vitellogenesis, oogenesis, reproduction, microbiomes

## Abstract

**Simple Summary:**

The abundance of insects indicates that they are one of the most adaptable forms of life on earth. Genetic, physiological, and biochemical plasticity and the extensive reproductive capacity of insects are some of the main reasons for such domination. The endocrine system has been known to regulate different stages of physiological and developmental processes such as metabolism, metamorphosis, growth, molting, and reproduction. However, in this review, we focus on those aspects of the endocrine system that regulate female insect reproduction. The proper understanding of the endocrine system will help us to better understand the insect reproductive system as well as to develop new strategies to control the insect pest population. The juvenile hormone analogs and molting hormone analogs have been widely used to control the insect pests. Such insect growth regulators are usually more specific and cause little harm to the beneficial organisms. Therefore, a proper understanding of these signaling pathways as well as their interaction with each other and other signaling pathways is very crucial. Further, the interaction of microbiome with the endocrine system is also discussed.

**Abstract:**

The proper synthesis and functioning of ecdysteroids and juvenile hormones (JHs) are very important for the regulation of vitellogenesis and oogenesis. However, their role and function contrast among different orders, and even in the same insect order. For example, the JH is the main hormone that regulates vitellogenesis in hemimetabolous insect orders, which include Orthoptera, Blattodea, and Hemiptera, while ecdysteroids regulate the vitellogenesis among the insect orders of Diptera, some Hymenoptera and Lepidoptera. These endocrine hormones also regulate each other. Even at some specific stage of insect life, they positively regulate each other, while at other stages of insect life, they negatively control each other. Such positive and negative interaction of 20-hydroxyecdysone (20E) and JH is also discussed in this review article to better understand the role of these hormones in regulating the reproduction. Therefore, the purpose of the present review is to deeply understand the complex interaction of endocrine hormones with each other and with the insulin signaling pathway. The role of microbiomes in the regulation of the insect endocrine system is also reviewed, as the endocrine hormones are significantly affected by the compounds produced by the microbiota.

## 1. Introduction

Reproductive physiology of insects includes all the physiological and behavioral processes from the development of the embryo to the production and oviposition of the fertile eggs [1]. Regulation of insect reproductive capacity coincides with the endocrine system [2]. The endocrine glands secrete hormones that are transported through the blood and act on tissues bearing specific receptors. However, endocrine regulation is very complex in insects and involves different types of hormones [3]. Therefore, in this review, we focus on the endocrine system in regulating female insect reproduction.

The prothoracic glands synthesize ecdysteroids after stimulation by the prothoracicotropic hormone (PTTH) and release them into the hemolymph [4], while the juvenile hormones (JHs) are secreted by a pair of endocrine glands behind the brain called the corpora allata (CA) [5]. The ecdysteroids are one of the major steroid hormones that play an essential part in regulating metamorphosis and larval molting [6]. However, such hormones are also vital and play crucial roles in regulating the reproductive physiology of insects [7]. Insects convert cholesterol into ecdysone and 20E (active metabolite) by the progression of some hydroxylation and oxidation steps. Such conversions are achieved by the involvement of cytochrome P450 enzymes encoded by Halloween genes [8]. During embryogenesis, the ecdysteroids are also maternally incorporated into the developing oocytes as conjugated ecdysteroids. Maternally deposited ecdysteroids then regulate a variety of cellular processes, which are vital for embryonic development. In *Bombyx mori*, the ecdysone oxidase was reported to be present in the cytoplasm throughout the yolk granules of the oocyte, and responsible for catalyzing 20E to 3-dehydroecdysone (3DE) through encoding an enzyme. Downregulation of *BmEO* by RNAi resulted in a significantly lower titer of 20E and hatching rate [9]. Meanwhile, during early embryogenesis, ecdysteroid-phosphate phosphatase (EPPase) converts the conjugated ecdysteroid into 20-hydroxyecdysone (20E) [10]. Mating-induced increased titer of 20E, in the hemolymph and ovaries of *Drosophila melanogaster*, results in increased expression of ecdysone-induced protein 75B (Eip75B) [11].

In different insects, both ecdysteroids and JHs regulate female insect reproduction in different ways. Among Lepidoptera, both 20E and JH control the female reproduction. However, they have a different role in the reproductive process like vitellogenesis and oogenesis among different insect species. For example, in *Helicoverpa armigera* and *Manduca sexta*, the JH has been known to significantly regulate female reproduction, while in *B. mori*, the egg development is mainly controlled by ecdysteroids [12]. Similarly, JHs are necessary for the proper synthesis of Vg in the fat body, while 20E signaling is vital for the ovarian development processes in *Tribolium castaneum* [13,14,15]. These internal regulatory factors are involved in oogenesis and embryonic development [16]. Therefore, we can say that endocrine hormones also regulate and affect each other. Thus, the proper understanding of these interlinked signaling pathways is crucial. Owing to advances in molecular biology, genomics, and bioinformatics, significant advancement has been accomplished in understanding the molecular channels that govern female insect reproduction. However, the proper interaction of these pathways with each other is very complex, and so here, we try to explain not only recent advances in understanding the role of ecdysteroids and JHs, but also their interaction together with the insulin signaling pathway and with microbiota.

## 2. 20-Hydroxyecdysone Regulated Reproduction in Insects

The ecdysteroids’ biosynthesis and signaling were found to be vital for the reproduction and longevity of adult insects [17]. The 20E produces its effects through binding with a heterodimer receptor. This receptor consists of the ecdysone receptor (EcR) and ultra-spiracle (USP) [18,19]. After binding with the 20E, the heterodimer complex interacts with the E response element (EcRE) [20,21], which later activates the early genes (broad complex (BrC, *E*74, and *E*75). *E*75 is a primary response gene, while *HR*3 is a secondary response gene [22]. Twenty-one nuclear receptors (NRs) were identified from the *Bacterocera dorsalis* [23], while Halloween genes encode for the enzymes (like cytochrome P450) necessary for catalyzing the last step of the ecdysteroid biosynthesis. In *Schistocerca gregaria*, *shade* (a Halloween gene) was found to encode 20-hydroxylase, which in turn catalyzed the conversion of 20E from ecdysone (E) [24]. However, the role of the *phantom* in ecdysteroid biosynthesis was also evaluated in *S. gregaria* [25]. Knockdown of EcR receptor (EcR-A) resulted in reduced expression of *Bombyx doublesex* (*Bmdsx*) in the embryos of both males and females. *Bmdsx* is responsible for the sexual differentiation of *B. mori*. Thus, ecdysone signaling is indirectly involved in the sexual differentiation of *B. mori* by affecting the expressions of *Bmdsx* [26]. Further, different points of oogenesis are also known to be controlled by more than 30 ecdysone responsive genes. Another Halloween gene, *spook* (spo), is found in the ovary of *D. melanogaster*. This gene encodes Cyp307A1 and is vital for the ecdysone biosynthetic pathway in the *Diptera* [27]. An increase in the ovary ecdysteroidogenic hormone (OEH) was found after blood-feeding in *Aedes aegypti* [28]. However, in *D. melanogaster*, the FLP-out system was used to knockdown the expressions of EcR and *E*75 from the escort cells, and the results indicated a decreased number of 16-cell cysts, meiotic entry, and follicle formation [29]. 20E also maintained the GSCs through *E*78 by controlling niche assembly [30]. These results suggested that the 20E is necessary for regulating different biological aspects of insect life [31].

In *Anopheles gambiae*, male mosquitoes transfer 20E to the female through seminal fluid, which results in the change of female behavior. Such transfer of 20E is essential in promoting postmating processes, because virgin females cannot induce such processes after blood feeding. Further when the sexual transfer of 20E is impeded by inhibiting its biosynthesis and partial inactivation in the males, then the oviposition is significantly reduced [32]. While in the case of Lepidoptera, the adults do not feed on rich protein sources needed for egg production [33]. In *Spodoptera litura*, mating results in the reduction of immunity, which in turn favors reproduction by saving the limited resources to support the high energy needed in reproduction (egg maturation and oviposition). Thus, Lepidoptera species can be a good a model to study mating-induced regulation in reproduction [34].

However, exogenous 20E negatively affected the reproduction of *Plutella xylostella*. Presence of exogenous 20E on the leaves of the host plant repelled the female. Female adults fed on exogenous 20E also displayed decreased fecundity. Further, the adult longevity and the development of eggs were also reduced [35].

### Interaction of 20E with JHs and Insulin Signaling Pathway

Proper balance between endocrine hormones is crucial for egg development. In female insects, JH levels significantly depend on diet and mating. An increase in the JH level upregulates the expression of yolk protein genes in the female of *T. castaneum*, *Blattella germanica*, and *B. dorsalis*. High levels of JH increase uptake of the vitellogenin in the oocytes, while a high 20E titer results in the resorption of vitellogenic eggs [36]. However, such a pattern of JH/20E signaling contrasts among different insects. For example, in mosquitoes, both 20E and JH are required for the oogenesis, but 20E is more essential for the regulation of vitellogenin eggs than JH [37]. 20E also controls the expression of ecdysis triggering hormone (ETH). Both 20E and ETH play an essential role in the reproductive success of insects. However, in *Colaphellus bowringi*, 20E and ETH also control the photoperiodic reproductive diapause. The 20E deficiency not only results in the decreased level of ETH, but also reduced the production of JH, while ETH knockdown decreased the mRNA levels of Vg1, Vg2, and of JH biosynthetic genes. Injected dsRNA of Met, EcR, ETH, and ETHR remarkably decreased the ovarian-specific expression profiles of Halloween genes (*Spo*, *Phm*, *Dib*, *Sad*, and *Shd*), in the long-day treated females. Exogenous treatment of both 20E and ETH peptides induced the vitellogenesis, and thus rescued the ovary development. Further, reduced expression of 20E, ETH, and JH results in the lipid accumulation. However, 20E might also repress lipid accumulation and could be independent of JH signaling. Therefore, it was suggested that the 20E controls the photoperiodic reproductive diapause of *C. bowringi* in both JH-dependent and JH-independent manners [38]. ETH plays a crucial role in the maintenance of juvenile hormone acid methyltransferase (JHAMT), which in turn is required for the normal production of JHs, vitellogenesis, and reproduction [39]. The injected dsRNA of ETH and ETHR in female adults of *B. dorsalis* resulted in reduced expression of JHAMT, Vg2, and JHs. Moreover, injection of 20E or methoprene rescued normal egg production [40].

**Figure 1:**
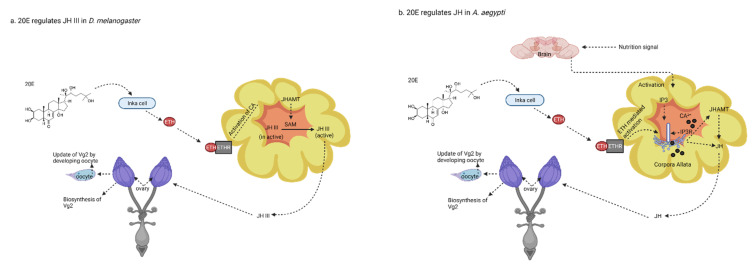
20E control of female reproduction through regulating juvenile hormones’ (JHs) production. (**a**) 20-hydroxyecdysone (20E) regulates JH III in the fruit fly. In the fruit fly, 20E not only regulates the synthesis and release of ecdysis triggering hormone (ETH) from the Inka cells, but also regulates the expressions of ETHR-B. Later, hemolymph released ETH binds with ETHR-B and activated the corpora allata (CA). However, in the CA, ETH also acts as an allatotropin and increase the activity of juvenile hormone acid methyltransferase (JHAMT). JHAMT regulates the biosynthesis of JHs by converting the inactive JHA III to active JH III in the presence of S-adenosyl methionine (SAM); however, CA released active JH III later regulates biosynthesis of vitellogenin in the ovary. Therefore, it was concluded that the 20E controls the female reproduction, ovary growth, and oocyte maturation by regulating the JHs. (**b**) 20E regulates JHs in the *A. aegypti*. In *A. aegypti*, in addition to hormones produced in the ovary, the brain also stimulates the CA to synthesis JHs, after sensing the nutritional signals. However, it was also observed that the ETH controls the JHAMT and JHs’ activity by mobilizing calcium from the endoplasmic reticulum stores, through the IP3 receptor.

In fruit fly, JHAMT converts JHA III to the active JH III by transferring the methyl group from S-adenosyl-L-methionine (SAM) to the carboxyl group of JH acids (Figure 1) [41]. The nutritional signals modulate the BR-C isoforms expressions in *Drosophila* eggs. Under starved conditions, these nutritional signals upregulated the BR-C Z2 and Z3 expressions, which in turn suppressed the *E*75*B* and overexpressed *E75A*. This overexpression of *E*75*A* caused the apoptosis of nurse cells at stages 8 and 9 in the egg chamber [42]. Mutation in EcR also reduced the expression of low-density lipoprotein receptor, LpR2, which caused the deficiency of lipids’ accumulation in the oocytes at stage 10 [43]. While in the adult stage of *D. melanogaster*, 20E increases the ETH production and control JHs’ biosynthesis to regulate reproduction [39]. Further, PTTH increases expression of the Halloween genes *Spook, Neverland*, *Disembodied*, and *Phantom* (JH-dependent), but not of *Shadow* and *Shroud* (JH-independent) [44]. However, in the adult mosquitoes, ETH controls the activity of JHAMT and JHs through the mobilization of endoplasmic reticulum Ca^2+^ stores. The inhibition of the IP3 receptor reduces ETH-dependent increase in the activity of JHAMT and JHs, respectively (Figure 1) [45]. However, the injection of 20E not only inhibited the JHMAT transcription, but also inhibited Vgs and VgR expressions, in the *Periplaneta americana* [46]. Meanwhile, in *B. mori*, insulin and bombyxin (insulin-like hormone) directly stimulate the prothoracic glands. Both insulin and PTTH increase the phosphorylation of Akt and stimulate the ecdysteroid secretion [47].

## 3. Juvenile Hormone Regulated Reproduction in Insects

The role of juvenile hormones in controlling insect metamorphosis and reproduction is of great importance [7]. Juvenile hormone regulates insect reproduction through its receptor Methoprene-tolerant (Met), which dimerizes with another bHLH-PAS protein Taiman (Tai). In the nucleus, the receptor complex of Tai and Met interacts with the target gene promoter DNA element, also known as JHs’ response element (JHREs) [12]. Tai did not bind with the JHs, but it is the JH-Met interaction that triggered the Met and Tai dimerization [48]. The nuclear import of Met is a crucial step in JH mediated signaling pathway. A chaperone protein, Hsp83, was recognized as JHRR-bound nuclear protein and was required for the JH-induced Kr-h1 expression [49]. A tetratricopeptide repeat (TPR) domain of Nup358 also interacts with Hsp83 and is crucial for nuclear localization of Met [50].

After the identification of Met as a JHs’ receptor, a regulatory model was also developed that involved JH-Met-Kr-h1 [51]. Kr-h1 knockdown resulted in the depletion of Met, which in turn clears the role of Kr-h1 in insect reproduction [7]. In *B. dorsalis*, the treatment of dsMet significantly reduced the expressions of BdKr-h1, BdMet, BdVg1, and BdVg2. Over 50% reduction in expressions of BdKr-h1, BdVg1 and BdVg2 were observed after 72 h treatment of dsKr-h1 [52]. The kr-h1 RNAi was also responsible for a 30% reduction of Vg expression in the female adults of *T. castaneum* [53]. In *Locusta migratoria*, *H. armigera*, and *Nilaparvata lugens*, suppression of Kr-h1 decreased the egg production by reducing the Vg expression, oocyte maturation, and ovarian development [54,55]. RNAi mediated silencing of Met not only blocked the ovary development, but also suppressed *Vg* gene expressions in *Pyrrhocoris apterus* fat body [56]. When the SgMet was knocked down in the *S. gregaria*, using RNAi, it was then observed that this insect never enters the previtellogenic stage. The ovaries of such treated insects were found to be arrested. Further, the mRNA level of kr-h1 was decreased by 88% and 73% in CA and fat body, respectively. Therefore, the JHs’ receptor Met is found to be necessary for the vitellogenesis, ovary maturation, and accessory ecdysteroid biosynthesis. Delayed copulation behavior is also found to be associated with the knockdown of SgMet. Further, a notable decrease in insulin-related peptides was also observed against dsSgMet-treated female adults of *S. gregaria* [57]. In cockroaches and locusts, JHs play a vital role in triggering the *Vg* genes. Met knockdown was responsible for the lower number of egg deposition. While in female adult mosquitoes, Met/Tai complex, a transcription factor, stimulates Kr-h1 expressions, which in turn promotes vitellogenesis [58]. In *Diploptera punctata*, ovarian growth was blocked owing to the silencing of Met, which in turn was responsible for a remarkable decrease in the size of developing eggs [59,60]. In *Reticulitermes speratus*, the Japanese termite, increase in the JH titers initiates Vg synthesis and results in the development of neotenic reproductivity [61]. Meanwhile, in *D. melanogaster*, JH regulates female mating and pheromone production [62]. In *A. aegypti* adult female mosquitoes, the expression of large number of genes is regulated by JH [63], while AaKrh1 knockdown significantly decreases egg production after blood feeding [55]. In *C. lectularius*, common bed bug, silencing of Kr-h1 does not decrease the number of oviposited eggs; however, it significantly affected the hatching of eggs [13]. In *H. armigera*, knockdown of HaKr-h1 also decreased the transcription of vitellogenin and interrupted oocyte maturation [64]. For *Grapholita molesta*, silencing of GmKr-h1 increased preoviposition period and decreased fecundity [65]. The queens of *Vespula vulgaris* releases honest signals to change the fertility status of subordinate workers, so their workers become reproductively repressed and help in colony. However, such signals are found to be controlled by endocrine hormones. To test if JH is the main hormone that maintains such fertility and fertility signaling, the workers were treated with JH analogue (methoprene) and JH inhibitor (precocene). The results showed that the oocyte size was increased after treatment with the methoprene; nevertheless, oocyte size was decreased against precocene. Thus, JH affects both fertility and fertility signaling in workers [66].

Meanwhile, JH also negatively affects the reproduction in some insects. Both *Streblognathus* and *Diacamma* have queenless societies. Reduction of JH titer in gamergates corresponds with high individual ranks within the hierarchy. As the alpha is responsible for the reproduction of offspring, JH treatment of such individual results in the loss of its reproductive status [67]. In *Dinoponera quadriceps*, JH regulates the female reproduction by affecting the ovary development. The ants with increased JH levels develop reduced ovarioles, which in turn decreased their reproductive potential by reducing the number of vitellogenic eggs [68]. 20E treatment of *Pteromalus puparum*, endoparasitic wasp, promoted *Vg gene* expression, while JH application resulted in the reduction of Vg mRNA levels [69]. However, knockdown of Kr-h1 did not affect the fecundity and Vg expressions of *C. lectularius* [32].

### Interaction of JHs with 20E and Insulin Signaling Pathway

Many aspects of reproduction, including vitellogenesis and oogenesis, are regulated by JH and 20E [70,71]. Protein 93F (E93) is an ecdysone induced protein that works as an adult specifier gene. Kr-h1 binding site (KBS) was identified in the E93 promoter region, and it was further observed that the JH-inducible Kr-h1 repressed the transcription of E93 through the direct binding with KBS. Additionally, a CtC motif was also identified in Kr-h1, which has been found necessary for the transcriptional repression of E93 [72]. The JHs also increased the Vg expressions by increasing the production of ILP2 [73].

In *Drosophila*, an increase in JHs’ level was observed under the influence of stress conditions, which in turn resulted in the accumulation of eggs and oviposition seize. However, increase in 20E resulted in breakdown of oocytes, which ultimately reduced fecundity. Therefore, the proper balance between JHs and 20E is necessary for the normal development of oogenesis [74]. However, during the previtellogenic phase, the JHs are involved with the fat body changes, which in turn makes the fat body sensitive to the signals that induce vitellogenesis [75].

In *T. castaneum*, JH regulates the expression of *Vg* gene in fat body, while 20E controls the synthesis of Vg by its effect on ovarian development and oocyte maturation [14,53]. In addition, JH also prompts Vg synthesis by controlling the expression of ILPs [73]. The JHs modulate the Vg expressions through an insulin-like peptide signaling pathway in the *T. castaneum*. Both JHs and feeding are found to be required for the proper synthesis of Vg in the fat body, while the JHs’ signaling pathway acted via Met and increased the production of ILP2. Feeding triggered the production of ILP3, and later the insulin like peptides stimulated the phosphorylation of AKT resulted in the FOXO phosphorylation and ultimately its depletion from the nucleus. The depletion of FOXO later allowed the expression of the *Vg* gene. Therefore, these results suggested that the JHs modulated the Vg expressions through the insulin-like peptide signaling pathway, which ultimately affect FOXO localization in the fat body. The JHs also indirectly regulate the vitellogenesis by inducing the production of insulin-IGF, which in turn activates IIS [73]. Meanwhile, in the cockroaches, including *B. germanica* and *P. americana*, JHs’ biosynthesis is promoted by insulin-IGF signaling (IIS) [76,77].

## 4. Microbiomes and Endocrine System

Insects harbor different microbial communities that affect their biology. However, in this review, we specifically focused on the regulation of insect reproduction by the interaction of microbiomes and the endocrine system. Both endocrine hormones and microbiota influence each other. Endocrine hormones affect the metabolism of microbiota by a number of different channels [78]. Reproductive microbiome affects the reproductive fitness of both male and female insects. The microbiomes significantly affect the reproductive system of insects, either by affecting the evolution of reproductive organs and or by producing their effect on sexual selection [79]. Reproductive organs of female harbor microorganisms that are transferred to their partner during copulation and even to their offspring [80]. Female copulation might also respond to microbial load. Fecundity of *Anopheles gambiae* significantly reduced when the female was infected with *Serratiochelin* and *Pyochelin* [81]. A bacterium, *Candidatus Erwinia*, increases the female reproductive output of *Bactrocera oleae* by increasing the production of essential amino acids [82]. Meanwhile, in another fruit fly, *Ceratitis capitata*, a group of nitrogen-fixing bacteria from Enterobacteriaceae family supports reproduction [83].

**Figure 2:**
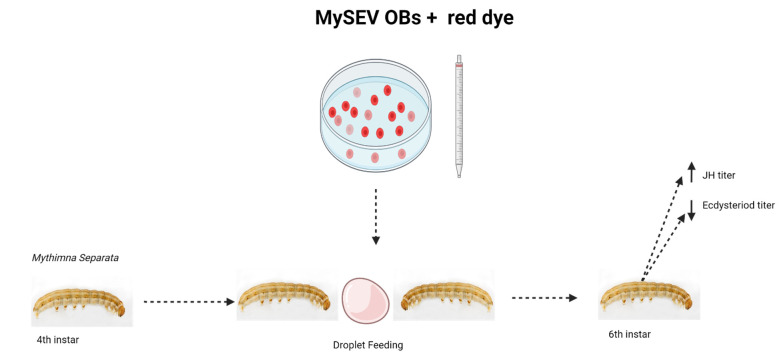
Regulation of endocrine hormones by the microbiota. The fourth instar of *M. separata* was inoculated with MySEV occlusion bodies, by the droplet feeding method. The results of liquid chromatography–MS analysis, against the sixth larval instar, revealed that the titer of juvenile hormone (JH) was significantly increased, whereas the titer of ecdysteroids was significantly decreased in the hemolymph of infected larvae.

The microbiota has also been used to produce adverse effects on the reproduction of insects. For example, *Beauveria bassiana* is an entomopathogenic fungus, and it affected different life stages of *Bemisia tabaci* [84]. Meanwhile, *Metarhizium anisopliae* significantly influenced the reproductive system of *Plutella xylostella*, by decreasing the life span and egg laying ability. A significant difference in the fecundity of treated insects was observed, where the mean number of laid eggs was reduced to 101.55 eggs/female as compared with control of 192.55 eggs/female [85]. In addition, plants also synthesize and store ecdysteroids, and the concentration of these phytoecdysteroids increases significantly with damage done by insects. Such phytoecdysteroids have been known to affect the adult reproduction by reducing the female fecundity. Further, both adults and larvae are also found to be repelled by such phytosteroids synthesized by plants [86]. The transcriptional regulatory molecular mechanism behind the overall improvement of physio-morphological attributes is also important in sustainability perspectives [87,88]. Syntech TrackSphere LC-100 locomotion compensator was used to analyze the locomotion of *Ostrinia nubilalis* larvae concerning to the odors from plants of genus Chenopodium. Except for *C. album* and *C. polyspermum*, the neonate larvae showed repellent behavior from all the tested phytosteroid-positive species [89], while treatment of 20E, a phytoecdysteroid, decreased the levels of soluble proteins in larvae of *T. castaneum* [90]. Owing to their significance, the microbiota is considered as important endocrine organ. The microbiota also synthesizes different compounds that circulate and affect the function of reproduction by regulating the endocrine system [91]. For example, a virus, betaentomopoxvirus, synthesizes sesquiterpenoid juvenile hormone through encoding JHs’ methyltransferase [92]. When the fourth instar larvae of *Mythimna separata* was treated with the entomopoxovirus, it reduced the titer of ecdysone and resulted in the death of sixth instar larvae. Further, JHs’ titer was increased in such treated insects (Figure 2) [93]. However, *Vairimorpha necatrix* infection against *Lacanobia oleracea* increased the JHs titer up to 10-fold [94]. Besides, modulating the hormone–receptor interactions, in the reproductive tissues, the metabolites of microbes can also modify the motility of gametes [95].

The microbiota transmits signals by electrochemical means, which also include ion channels [96]. However, it is still not clear how the microbiomes affect the insect endocrine system. Further studies are required to find out the type of molecules they make in order to better understand the role of microbiome in regulating adult insect’s reproduction, by regulating the endocrine system. The exact pathway of microbiota-hormonal signaling has not yet been explored. However, studies have proved that the changes in hormonal levels were associated with the presence of microbiota. Further, microbiota synthesizes hormones, responds to host hormone, and regulates the expression of host hormones [97].

In addition to the regulation of insect reproduction, the microbiota also regulates multiple functions for host health, including food digestion, displacement of pathogens, and synthesis of vitamin [98,99]. In vitro methods can also be used further to understand the interaction of microbiota with the endocrine system.

## 5. Conclusions and Future Prospects

In recent advances, researchers have been able to identify receptors of ecdysteroids and JHs, which helped us to better understand these signalling pathways in various insect orders. Such studies also explained the different events of reproduction among different insect orders. However, the interaction of these endocrine hormones between each other is very complex. For example, fruit flies belong to Diptera. An increase in the JHs’ level positively controls the egg development by increasing the vitellogenin uptake in the eggs. Meanwhile, the increase in the 20E titer negatively regulates the eggs’ development by resorption of vitellogenin. However, in mosquitoes, 20E has a major role in regulating the reproduction, where an increase in the 20E positively regulates the egg development. Thus, the function of these endocrine hormones contrasts among different species of the same insect order. Whereas among Lepidoptera, the function of these endocrine hormones also varies between different species. As adults of the tobacco cutworm feed on a diet with less amino acids contents, the mating supports the reproduction by decreasing the immune system to save the limited resources needed in reproduction. In addition, the endocrine hormone (JH) is also transferred from males to females, which helps in the egg development. On the other hand, in the diamondback moth, exogenous application of 20E results in the reduction of female fecundity. Recent findings also explained the role of these endocrine hormones in controlling the fertility of insects. However, such a complex interaction of these endocrine hormones is also necessary for regulating the reproduction of such insects.

The role of AA/TOR and insulin signaling pathways not only cleared the vitellogenin intake by the developing oocyte, but also explained its role in the biosynthesis of the JHs’ hormones and ecdysteroids. Little information is available regarding the proper understanding of these signaling pathways in less studied insects. Therefore, in the present review, we also focused on the role of endocrine hormones in such less studied insects. Further, the interaction of these endocrine hormones is also explained. However, the comparative contribution and the interaction of these signaling pathways with each other further require clarity.

When the insect comes into contact with the microorganism, a change in the titer of endocrine hormones is observed. Such changes in the endocrine hormone titer significantly affect their reproduction in several different ways. A significant increase in reproduction of the olive fruit fly is observed after treatment with *C. Erwinia*. Such bacteria increased the female reproductive output by increasing the production of essential amino acids. Female insects also transfer microorganisms to their partner as well as to their offspring. Therefore, in this review, we not only focused on the mechanism of transcriptional activation of 20E and JHs, but also discussed the complex interaction of these signaling pathways together with each other and with other signaling pathways (insulin signaling pathway). In addition, the role of microbiomes in regulating the endocrine hormones has been discussed. However, further studies should focus on the entire microbiome and the type of molecules they create in order to better understand their interaction with the endocrine hormones. As endocrine analogs are environmentally safe, the proper understanding of female endocrine system and reproductive system together with the microbiota will help us to control the insect pest more effectively.

## Data Availability

Not applicable.

## References

[1] Chiang R.G., Chiang J.A. (2017). Reproductive physiology in the blood feeding insect, Rhodnius prolixus, from copulation to the control of egg production. J. Insect Physiol..

[2] Kelstrup H.C., Hartfelder K., Nascimento F.S., Riddiford L.M. (2014). Reproductive status, endocrine physiology and chemical signaling in the Neotropical, swarm-founding eusocial wasp Polybia micans. J. Exp. Biol..

[3] Niwa R., Niwa Y.S. (2014). Enzymes for ecdysteroid biosynthesis: Their biological functions in insects and beyond. Biosci. Biotechnol. Biochem..

[4] Hentze J.L., Moeller M.E., Jørgensen A.F., Bengtsson M.S., Bordoy A.M., Warren J.T., Gilbert L.I., Andersen O., Rewitz K.F. (2013). Accessory gland as a site for prothoracicotropic hormone controlled ecdysone synthesis in adult male insects. PLoS ONE.

[5] Mirth C.K., Tang H.Y., Makohon-Moore S.C., Salhadar S., Gokhale R.H., Warner R.D., Koyama T., Riddiford L.M., Shingleton A.W. (2014). Juvenile hormone regulates body size and perturbs insulin signaling in Drosophila. Proc. Natl. Acad. Sci. USA.

[6] Ishimoto H., Kitamoto T. (2010). The steroid molting hormone Ecdysone regulates sleep in adult Drosophila melanogaster. Genetics.

[7] Song J., Wu Z., Wang Z., Deng S., Zhou S. (2014). Krüppel-homolog 1 mediates juvenile hormone action to promote vitellogenesis and oocyte maturation in the migratory locust. Insect Biochem. Mol. Biol..

[8] Uryu O., Ou Q., Komura-Kawa T., Kamiyama T., Iga M., Syrzycka M., Hirota K., Kataoka H., Honda B.M., King-Jones K. (2018). Cooperative control of ecdysone biosynthesis in Drosophila by transcription factors Séance, Ouija board, and Molting defective. Genetics.

[9] Wang C.-F., Zhang Z., Sun W. (2018). Ecdysone oxidase and 3-dehydroecdysone-3β-reductase contribute to the synthesis of ecdysone during early embryonic development of the silkworm. Int. J. Biol. Sci..

[10] Sonobe H., Yamada R. (2004). Ecdysteroids during early embryonic development in silkworm Bombyx mori: Metabolism and functions. Zool. Sci..

[11] Zipper L., Jassmann D., Burgmer S., Görlich B., Reiff T. (2020). Ecdysone steroid hormone remote controls intestinal stem cell fate decisions via the PPARγ-homolog Eip75B in Drosophila. elife.

[12] Roy S., Saha T.T., Zou Z., Raikhel A.S. (2018). Regulatory pathways controlling female insect reproduction. Annu. Rev. Entomol..

[13] Gujar H., Palli S.R. (2016). Juvenile hormone regulation of female reproduction in the common bed bug, Cimex lectularius. Sci. Rep..

[14] Parthasarathy R., Sheng Z., Sun Z., Palli S.R. (2010). Ecdysteroid regulation of ovarian growth and oocyte maturation in the red flour beetle, Tribolium castaneum. Insect Biochem. Mol. Biol..

[15] Wu Z., Yang L., He Q., Zhou S. (2021). Regulatory Mechanisms of Vitellogenesis in Insects. Front. Cell Dev. Biol..

[16] Liu S., Lucas K.J., Roy S., Ha J., Raikhel A.S. (2014). Mosquito-specific microRNA-1174 targets serine hydroxymethyltransferase to control key functions in the gut. Proc. Natl. Acad. Sci. USA.

[17] Schwedes C.C., Carney G.E. (2012). Ecdysone signaling in adult Drosophila melanogaster. J. Insect Physiol..

[18] Lenaerts C., Marchal E., Peeters P., Broeck J.V. (2019). The ecdysone receptor complex is essential for the reproductive success in the female desert locust, Schistocerca gregaria. Sci. Rep..

[19] Gauhar Z., Sun L.V., Hua S., Mason C.E., Fuchs F., Li T.-R., Boutros M., White K.P. (2009). Genomic mapping of binding regions for the Ecdysone receptor protein complex. Genome Res..

[20] Hill R.J., Billas I.M., Bonneton F., Graham L.D., Lawrence M.C. (2013). Ecdysone receptors: From the Ashburner model to structural biology. Annu. Rev. Entomol..

[21] King-Jones K., Thummel C.S. (2005). Nuclear receptors—A perspective from Drosophila. Nat. Rev. Genet..

[22] Mazina M.Y., Kocheryzhkina E., Nikolenko J., Krasnov A., Georgieva S., Vorobyeva N. (2017). Nuclear receptors EcR, Usp, E75, DHR3, and ERR regulate transcription of ecdysone cascade genes. Dokl. Biochem. Biophys..

[23] Yang P.-J., Chen E.-H., Song Z.-H., He W., Liu S.-H., Dou W., Wang J.-J. (2020). Molecular Characterization and Expression Profiling of Nuclear Receptor Gene Families in Oriental Fruit Fly, Bactrocera Dorsalis (Hendel). Insects.

[24] Marchal E., Verlinden H., Badisco L., Van Wielendaele P., Broeck J.V. (2012). RNAi-mediated knockdown of Shade negatively affects ecdysone-20-hydroxylation in the desert locust, Schistocerca gregaria. J. Insect Physiol..

[25] Marchal E., Badisco L., Verlinden H., Vandersmissen T., Van Soest S., Van Wielendaele P., Broeck J.V. (2011). Role of the Halloween genes, Spook and Phantom in ecdysteroidogenesis in the desert locust, Schistocerca gregaria. J. Insect Physiol..

[26] Matsushima D., Kasahara R., Matsuno K., Aoki F., Suzuki M.G. (2019). Involvement of Ecdysone Signaling in the Expression of the doublesex Gene during Embryonic Development in the Silkworm, Bombyx mori. Sex. Dev..

[27] Ono H., Rewitz K.F., Shinoda T., Itoyama K., Petryk A., Rybczynski R., Jarcho M., Warren J.T., Marqués G., Shimell M.J. (2006). Spook and Spookier code for stage-specific components of the ecdysone biosynthetic pathway in Diptera. Dev. Biol..

[28] Vogel K.J., Brown M.R., Strand M.R. (2015). Ovary ecdysteroidogenic hormone requires a receptor tyrosine kinase to activate egg formation in the mosquito Aedes aegypti. Proc. Natl. Acad. Sci. USA.

[29] Morris L.X., Spradling A.C. (2012). Steroid signaling within Drosophila ovarian epithelial cells sex-specifically modulates early germ cell development and meiotic entry. PLoS ONE.

[30] Ables E.T., Bois K.E., Garcia C.A., Drummond-Barbosa D. (2015). Ecdysone response gene E78 controls ovarian germline stem cell niche formation and follicle survival in Drosophila. Dev. Biol..

[31] Li Y., Ma Q., Cherry C.M., Matunis E.L. (2014). Steroid signaling promotes stem cell maintenance in the Drosophila testis. Dev. Biol..

[32] Gabrieli P., Kakani E.G., Mitchell S.N., Mameli E., Want E.J., Anton A.M., Serrao A., Baldini F., Catteruccia F. (2014). Sexual transfer of the steroid hormone 20E induces the postmating switch in Anopheles gambiae. Proc. Natl. Acad. Sci. USA.

[33] Di X., Liu J., Wu C., Yan B., Yu X., Yang M. (2020). Delayed mating with multiple partners decreases indexes of mating in female and male Spodoptera litura (Lepidoptera: Noctuidae). Environ. Entomol..

[34] Gao B., Song X.-Q., Yu H., Fu D.-Y., Xu J., Ye H. (2020). Mating-Induced Differential Expression in Genes Related to Reproduction and Immunity in Spodoptera litura (Lepidoptera: Noctuidae) Female Moths. J. Insect Sci..

[35] Sun L.J., Liu Y.J., Shen C.P. (2015). The effects of exogenous 20-hydroxyecdysone on the feeding, development, and reproduction of Plutella xylostella (Lepidoptera: Plutellidae). Fla. Entomol..

[36] Schwenke R.A., Lazzaro B.P., Wolfner M.F. (2016). Reproduction–immunity trade-offs in insects. Annu. Rev. Entomol..

[37] Hansen I.A., Attardo G.M., Rodriguez S.D., Drake L.L. (2014). Four-way regulation of mosquito yolk protein precursor genes by juvenile hormone-, ecdysone-, nutrient-, and insulin-like peptide signaling pathways. Front. Physiol..

[38] Guo S., Tian Z., Wu Q.-W., King-Jones K., Liu W., Zhu F., Wang X.-P. (2021). Steroid hormone ecdysone deficiency stimulates preparation for photoperiodic reproductive diapause. PLoS Genet..

[39] Meiselman M., Lee S.S., Tran R.-T., Dai H., Ding Y., Rivera-Perez C., Wijesekera T.P., Dauwalder B., Noriega F.G., Adams M.E. (2017). Endocrine network essential for reproductive success in Drosophila melanogaster. Proc. Natl. Acad. Sci. USA.

[40] Shi Y., Liu T.-Y., Jiang H.-B., Liu X.-Q., Dou W., Park Y., Smagghe G., Wang J.-J. (2019). The ecdysis triggering hormone system, via ETH/ETHR-B, is essential for successful reproduction of a major pest insect, Bactrocera dorsalis (Hendel). Front. Physiol..

[41] Niwa R., Niimi T., Honda N., Yoshiyama M., Itoyama K., Kataoka H., Shinoda T. (2008). Juvenile hormone acid O-methyltransferase in Drosophila melanogaster. Insect Biochem. Mol. Biol..

[42] Terashima J., Bownes M. (2006). E75A and E75B have opposite effects on the apoptosis/development choice of the Drosophila egg chamber. Cell Death Differ..

[43] Sieber M.H., Spradling A.C. (2015). Steroid signaling establishes a female metabolic state and regulates SREBP to control oocyte lipid accumulation. Curr. Biol..

[44] Moulos P., Alexandratos A., Nellas I., Dedos S.G. (2018). Refining a steroidogenic model: An analysis of RNA-seq datasets from insect prothoracic glands. BMC Genom..

[45] Areiza M., Nouzova M., Rivera-Perez C., Noriega F.G. (2014). Ecdysis triggering hormone ensures proper timing of juvenile hormone biosynthesis in pharate adult mosquitoes. Insect Biochem. Mol. Biol..

[46] Kamruzzaman A., Mikani A., Mohamed A.A., Elgendy A.M., Takeda M. (2020). Crosstalk among Indoleamines, Neuropeptides and JH/20E in Regulation of Reproduction in the American Cockroach, Periplaneta americana. Insects.

[47] Gu S.-H., Lin J.-L., Lin P.-L., Chen C.-H. (2009). Insulin stimulates ecdysteroidogenesis by prothoracic glands in the silkworm, Bombyx mori. Insect Biochem. Mol. Biol..

[48] Wang Z., Yang L., Song J., Kang L., Zhou S. (2017). An isoform of Taiman that contains a PRD-repeat motif is indispensable for transducing the vitellogenic juvenile hormone signal in Locusta migratoria. Insect Biochem. Mol. Biol..

[49] He Q., Wen D., Jia Q., Cui C., Wang J., Palli S.R., Li S. (2014). Heat shock protein 83 (Hsp83) facilitates methoprene-tolerant (Met) nuclear import to modulate juvenile hormone signaling. J. Biol. Chem..

[50] He Q., Zhang Y., Zhang X., Xu D., Dong W., Li S., Wu R. (2017). Nucleoporin Nup358 facilitates nuclear import of Methoprene-tolerant (Met) in an importin β-and Hsp83-dependent manner. Insect Biochem. Mol. Biol..

[51] Huang J.-H., Lozano J., Belles X. (2013). Broad-complex functions in postembryonic development of the cockroach Blattella germanica shed new light on the evolution of insect metamorphosis. Biochim. Biophys. Acta (BBA) Gen. Subj..

[52] Yue Y., Yang R.-L., Wang W.-P., Zhou Q.-H., Chen E.-H., Yuan G.-R., Wang J.-J., Dou W. (2018). Involvement of Met and Kr-h1 in JH-mediated reproduction of female Bactrocera dorsalis (Hendel). Front. Physiol..

[53] Parthasarathy R., Sun Z., Bai H., Palli S.R. (2010). Juvenile hormone regulation of vitellogenin synthesis in the red flour beetle, Tribolium castaneum. Insect Biochem. Mol. Biol..

[54] Lin X., Yao Y., Wang B. (2015). Methoprene-tolerant (Met) and Krüpple-homologue 1 (Kr-h1) are required for ovariole development and egg maturation in the brown plant hopper. Sci. Rep..

[55] Ojani R., Fu X., Ahmed T., Liu P., Zhu J. (2018). Krüppel homologue 1 acts as a repressor and an activator in the transcriptional response to juvenile hormone in adult mosquitoes. Insect Mol. Biol..

[56] Smykal V., Bajgar A., Provaznik J., Fexova S., Buricova M., Takaki K., Hodkova M., Jindra M., Dolezel D. (2014). Juvenile hormone signaling during reproduction and development of the linden bug, Pyrrhocoris apterus. Insect Biochem. Mol. Biol..

[57] Gijbels M., Lenaerts C., Broeck J.V., Marchal E. (2019). Juvenile Hormone receptor Met is essential for ovarian maturation in the Desert Locust, Schistocerca gregaria. Sci. Rep..

[58] Wang J.-L., Saha T.T., Zhang Y., Zhang C., Raikhel A.S. (2017). Juvenile hormone and its receptor methoprene-tolerant promote ribosomal biogenesis and vitellogenesis in the Aedes aegypti mosquito. J. Biol. Chem..

[59] Marchal E., Hult E.F., Huang J., Pang Z., Stay B., Tobe S.S. (2014). Methoprene-tolerant (Met) knockdown in the adult female cockroach, Diploptera punctata completely inhibits ovarian development. PLoS ONE.

[60] Villalobos-Sambucaro M.J., Riccillo F.L., Calderón-Fernández G.M., Sterkel M., Diambra L.A., Ronderos J.R. (2015). Genomic and functional characterization of a methoprene-tolerant gene in the kissing-bug Rhodnius prolixus. Gen. Comp. Endocrinol..

[61] Saiki R., Gotoh H., Toga K., Miura T., Maekawa K. (2015). High juvenile hormone titre and abdominal activation of JH signalling may induce reproduction of termite neotenics. Insect Mol. Biol..

[62] Bilen J., Atallah J., Azanchi R., Levine J.D., Riddiford L.M. (2013). Regulation of onset of female mating and sex pheromone production by juvenile hormone in Drosophila melanogaster. Proc. Natl. Acad. Sci. USA.

[63] Zou Z., Saha T.T., Roy S., Shin S.W., Backman T.W., Girke T., White K.P., Raikhel A.S. (2013). Juvenile hormone and its receptor, methoprene-tolerant, control the dynamics of mosquito gene expression. Proc. Natl. Acad. Sci. USA.

[64] Zhang W.N., Ma L., Liu C., Chen L., Xiao H.J., Liang G.M. (2018). Dissecting the role of Krüppel homolog 1 in the metamorphosis and female reproduction of the cotton bollworm, Helicoverpa armigera. Insect Mol. Biol..

[65] Zhang J., Liu X., Liu Y., An Y., Fang H., Michaud J., Zhang H., Li Y., Zhang Q., Li Z. (2019). Molecular characterization of primary juvenile hormone responders Methoprene-tolerant (Met) and Krüppel homolog 1 (Kr-h1) in Grapholita molesta (Lepidoptera: Tortricidae) with clarification of their roles in metamorphosis and reproduction. J. Econ. Entomol..

[66] Oi C.A., Ferreira H.M., da Silva R.C., Bienstman A., Nascimento F.S.d., Wenseleers T. (2021). Effects of juvenile hormone in fertility and fertility-signaling in workers of the common wasp Vespula vulgaris. PLoS ONE.

[67] Brent C., Peeters C., Dietmann V., Crewe R., Vargo E. (2006). Hormonal correlates of reproductive status in the queenless ponerine ant, Streblognathus peetersi. J. Comp. Physiol. A.

[68] Cuvillier-Hot V., Lenoir A., Peeters C. (2004). Reproductive monopoly enforced by sterile police workers in a queenless ant. Behav. Ecol..

[69] Dong S.-Z., Ye G.-Y., Guo J.-Y., Hu C. (2009). Roles of ecdysteroid and juvenile hormone in vitellogenesis in an endoparasitic wasp, Pteromalus puparum (Hymenoptera: Pteromalidae). Gen. Comp. Endocrinol..

[70] Swevers L. (2019). An update on ecdysone signaling during insect oogenesis. Curr. Opin. Insect Sci..

[71] Santos C.G., Humann F.C., Hartfelder K. (2019). Juvenile hormone signaling in insect oogenesis. Curr. Opin. Insect Sci..

[72] Kayukawa T., Jouraku A., Ito Y., Shinoda T. (2017). Molecular mechanism underlying juvenile hormone-mediated repression of precocious larval–adult metamorphosis. Proc. Natl. Acad. Sci. USA.

[73] Sheng Z., Xu J., Bai H., Zhu F., Palli S.R. (2011). Juvenile hormone regulates vitellogenin gene expression through insulin-like peptide signaling pathway in the red flour beetle, Tribolium castaneum. J. Biol. Chem..

[74] Gruntenko N., Rauschenbach I.Y. (2008). Interplay of JH, 20E and biogenic amines under normal and stress conditions and its effect on reproduction. J. Insect Physiol..

[75] Zhu J., Chen L., Raikhel A.S. (2003). Posttranscriptional control of the competence factor βFTZ-F1 by juvenile hormone in the mosquito Aedes aegypti. Proc. Natl. Acad. Sci. USA.

[76] Abrisqueta M., Süren-Castillo S., Maestro J.L. (2014). Insulin receptor-mediated nutritional signalling regulates juvenile hormone biosynthesis and vitellogenin production in the German cockroach. Insect Biochem. Mol. Biol..

[77] Li S., Zhu S., Jia Q., Yuan D., Ren C., Li K., Liu S., Cui Y., Zhao H., Cao Y. (2018). The genomic and functional landscapes of developmental plasticity in the American cockroach. Nat. Commun..

[78] Aguilera M., Gálvez-Ontiveros Y., Rivas A. (2020). Endobolome, a New Concept for Determining the Influence of Microbiota Disrupting Chemicals (MDC) in Relation to Specific Endocrine Pathogenesis. Front. Microbiol..

[79] Rowe M., Veerus L., Trosvik P., Buckling A., Pizzari T. (2020). The reproductive microbiome: An emerging driver of sexual selection, sexual conflict, mating systems, and reproductive isolation. Trends Ecol. Evol..

[80] Otti O. (2015). Genitalia-associated microbes in insects. Insect Sci..

[81] Ganley J.G., Pandey A., Sylvester K., Lu K.-Y., Toro-Moreno M., Rütschlin S., Bradford J.M., Champion C.J., Böttcher T., Xu J. (2020). A Systematic Analysis of Mosquito-Microbiome Biosynthetic Gene Clusters Reveals Antimalarial Siderophores that Reduce Mosquito Reproduction Capacity. Cell Chem. Biol..

[82] Ben-Yosef M., Pasternak Z., Jurkevitch E., Yuval B. (2014). Symbiotic bacteria enable olive flies (Bactrocera oleae) to exploit intractable sources of nitrogen. J. Evol. Biol..

[83] Malacrinò A., Campolo O., Medina R.F., Palmeri V. (2018). Instar-and host-associated differentiation of bacterial communities in the Mediterranean fruit fly Ceratitis capitata. PLoS ONE.

[84] Zafar J., Freed S., Khan B.A., Farooq M. (2016). Effectiveness of Beauveria bassiana against cotton whitefly, Bemisia tabaci (Gennadius)(Aleyrodidae: Homoptera) on different host plants. Pak. J. Zool..

[85] Zafar J., Shoukat R.F., Zhang Y., Freed S., Xu X., Jin F. (2020). Metarhizium Anisopliae Challenges Immunity and Demography of Plutella xylostella. Insects.

[86] Schäpers A., Petrén H., Wheat C.W., Wiklund C., Friberg M. (2017). Female fecundity variation affects reproducibility of experiments on host plant preference and acceptance in a phytophagous insect. Proc. R. Soc. B Biol. Sci..

[87] Javed T., Shabbir R., Ali A., Afzal I., Zaheer U., Gao S.-J. (2020). Transcription factors in plant stress responses: Challenges and potential for sugarcane improvement. Plants.

[88] Shabbir R., Javed T., Afzal I., Sabagh A.E., Ali A., Vicente O., Chen P. (2021). Modern Biotechnologies: Innovative and Sustainable Approaches for the Improvement of Sugarcane Tolerance to Environmental Stresses. Agronomy.

[89] Piesik D., Rochat D., Bocianowski J., Marion-Poll F. (2018). Repellent activity of plants from the genus Chenopodium to Ostrinia nubilalis larvae. Plant Prot. Sci..

[90] Ajaha A., Bouayad N., Aarab A., Rharrabe K. (2019). Effect of 20-hydroxyecdysone, a phytoecdysteroid, on development, digestive, and detoxification enzyme activities of Tribolium castaneum (Coleoptera: Tenebrionidae). J. Insect Sci..

[91] Clarke G., Stilling R.M., Kennedy P.J., Stanton C., Cryan J.F., Dinan T.G. (2014). Minireview: Gut microbiota: The neglected endocrine organ. Mol. Endocrinol..

[92] Takatsuka J., Nakai M., Shinoda T. (2017). A virus carries a gene encoding juvenile hormone acid methyltransferase, a key regulatory enzyme in insect metamorphosis. Sci. Rep..

[93] Nakai M., Kinjo H., Takatsuka J., Shiotsuki T., Kamita S.G., Kunimi Y. (2016). Entomopoxvirus infection induces changes in both juvenile hormone and ecdysteroid levels in larval Mythimna separata. J. Gen. Virol..

[94] Down R.E., Bell H.A., Bryning G., Kirkbride-Smith A.E., Edwards J.P., Weaver R.J. (2008). Infection by the microsporidium Vairimorpha necatrix (Microspora: Microsporidia) elevates juvenile hormone titres in larvae of the tomato moth, Lacanobia oleracea (Lepidoptera: Noctuidae). J. Invertebr. Pathol..

[95] Williams C.L., Garcia-Reyero N., Martyniuk C.J., Tubbs C.W., Bisesi J.H. (2020). Regulation of endocrine systems by the microbiome: Perspectives from comparative animal models. Gen. Comp. Endocrinol..

[96] Prindle A., Liu J., Asally M., Ly S., Garcia-Ojalvo J., Süel G.M. (2015). Ion channels enable electrical communication in bacterial communities. Nature.

[97] Freestone P.P., Sandrini S.M., Haigh R.D., Lyte M. (2008). Microbial endocrinology: How stress influences susceptibility to infection. Trends Microbiol..

[98] Neish A.S. (2014). Mucosal immunity and the microbiome. Ann. Am. Thorac. Soc..

[99] Trompette A., Gollwitzer E.S., Yadava K., Sichelstiel A.K., Sprenger N., Ngom-Bru C., Blanchard C., Junt T., Nicod L.P., Harris N.L. (2014). Gut microbiota metabolism of dietary fiber influences allergic airway disease and hematopoiesis. Nat. Med..

